# Machine learning–based prognostic modeling for locally advanced non-small cell lung cancer treated with immuno-radiotherapy

**DOI:** 10.3389/fphar.2025.1723874

**Published:** 2025-12-01

**Authors:** Simin Lu, Xin Zheng, Yi Wang, Yafei Li, Xuan Yu, Yan Zhang

**Affiliations:** 1 Department of Oncology, Luzhou People’s Hospital, Luzhou, China; 2 Department of Nasopharyngeal Carcinoma, Sun Yat-sen University Cancer Center, State Key Laboratory of Oncology in South China, Guangdong Key Laboratory of Nasopharyngeal Carcinoma Diagnosis and Therapy, Guangdong Provincial Clinical Research Center for Cancer, Sun Yat-sen University Cancer Center, Guangzhou, China

**Keywords:** non-small cell lung cancer, chemoradiotherapy, immunotherapy, artificial intelligence, random survival forest

## Abstract

**Background:**

Patients with locally advanced non-small cell lung cancer (NSCLC) who undergo concurrent chemoradiotherapy (CCRT) followed by consolidation immunotherapy show heterogeneous survival outcomes. Accurate prognostic prediction remains a major challenge in clinical practice. This study aimed to develop machine learning models to enhance personalized outcome prediction and guide precision immuno-radiotherapy.

**Methods:**

A total of 219 patients with locally advanced NSCLC were retrospectively enrolled. All patients received standard CCRT followed by consolidation immunotherapy. Prognostic variables were first selected using least absolute shrinkage and selection operator (LASSO) regression. A multivariate Cox proportional hazards model and a random survival forest (RSF) model were then constructed in the training cohort and validated in the independent cohort.

**Results:**

LASSO regression identified four prognostic variables: Age, T stage, Stage, and Pathology. Multivariate Cox analysis confirmed Stage and Pathology as independent predictors of OS. The Cox model achieved a C-index of 0.62 and Area Under the Receiver Operating Characteristic Curve (AUC-ROC) of 0.748 and 0.736 for 1-and 2-year OS in the validation cohort. The RSF model demonstrated higher predictive accuracy, with a C-index of 0.67 and AUC-ROC of 0.79 and 0.78 for 1-and 2-year OS, respectively. Variable importance analysis indicated that Stage and Pathology were the most influential factors. Based on RSF-derived risk scores, patients were stratified into high-and low-risk groups, and the high-risk group showed significantly poorer survival.

**Conclusion:**

The RSF model demonstrated improved performance compared to the conventional Cox model in predicting survival and stratifying risk among patients with locally advanced NSCLC undergoing CCRT and consolidation immunotherapy.

## Background

Lung cancer remains the leading cause of cancer-related mortality worldwide, accounting for approximately 1.8 million deaths annually (Bade and Dela Cruz, 2020). Among all histological types, non-small cell lung cancer (NSCLC) constitutes nearly 85% of cases (Nasim et al., 2019). Despite substantial advances in diagnostic imaging and systemic therapy, a considerable proportion of patients are still diagnosed at locally advanced stages (stage III), when the tumor burden is high and curative resection is often infeasible (Chen et al., 2022). The global burden of NSCLC continues to rise, particularly in regions with persistent exposure to smoking, environmental pollution, and occupational carcinogens, posing a major challenge to public health systems (Molina et al., 2008).

For patients with locally advanced NSCLC who lose the opportunity for surgical intervention, concurrent chemoradiotherapy (CCRT) remains the cornerstone of treatment (Garassino et al., 2022). However, disease control after standard CCRT is suboptimal, with a high risk of locoregional recurrence and distant metastasis. The advent of immune checkpoint inhibitors (ICIs), especially programmed death-1 (PD-1) and programmed death-ligand 1 (PD-L1) antibodies, has revolutionized the therapeutic landscape. Consolidation immunotherapy following definitive chemoradiotherapy has demonstrated significant survival benefits and has become a new standard of care (Liu et al., 2025; Ricciuti et al., 2025). Nonetheless, the clinical response to immuno-radiotherapy varies markedly across patients due to tumor heterogeneity, dynamic immune microenvironments, and radiotherapy-induced immune modulation (Moradi et al., 2025; Amat et al., 2025). Reliable biomarkers and individualized prediction models for treatment efficacy remain urgently needed.

Recently, machine learning has shown great potential in improving clinical decision-making for cancer therapy (Su et al., 2025). By leveraging large-scale clinical, imaging, and treatment data, machine learning algorithms can capture complex nonlinear relationships and identify subtle patterns beyond traditional statistical methods (Li et al., 2025). In the context of immuno-radiotherapy, these algorithms can help predict treatment response, optimize radiotherapy parameters, and stratify patients according to potential benefit. Furthermore, integrating machine learning into clinical workflows allows dynamic, data-driven evaluation and facilitates personalized therapeutic strategies that could enhance precision and efficiency in managing locally advanced NSCLC (Li et al., 2024).

In summary, the integration of machine learning into the management of locally advanced NSCLC represents a promising step toward precision immuno-radiotherapy. By identifying predictive factors and optimizing treatment planning, machine learning-based models may help overcome the limitations of empirical decision-making. Continued interdisciplinary collaboration among oncologists, radiologists, and data scientists is essential to translate these approaches into clinical practice, ultimately improving survival and quality of life for patients with locally advanced NSCLC.

## Methods

### Patients and ethical statement

A total of 219 patients with NSCLC were retrospectively enrolled from two tertiary hospitals.

The inclusion criteria were as follows:

1. histopathologically confirmed NSCLC;
2. locally advanced disease;
3. no evidence of distant metastasis on baseline imaging;
4. received concurrent chemoradiotherapy followed by consolidation immunotherapy;
5. complete follow-up information and survival outcome data available.

The exclusion criteria included:

1. incomplete clinicopathological or treatment data;
2. prior history of malignancy or concurrent other primary tumors;
3. radiotherapy course not completed or total dose <60 Gy;
4. loss to follow-up within 3 months after treatment.

This study was approved by the Ethics Committee of Luzhou People’s Hospital (22w202501016) and conducted in accordance with the principles of the Declaration of Helsinki. The requirement for informed consent was waived due to the retrospective nature of the study, and all patient data were anonymized prior to analysis.

### Treatment and follow-up

All patients received concurrent chemoradiotherapy (CCRT) followed by consolidation immunotherapy as the standard treatment protocol.

Overall survival (OS) was defined as the primary endpoint of this study. OS was defined as the time from the date of initial treatment to death from any cause or the last follow-up and was used as the primary endpoint of this study.

### AI-based model construction and validation

All enrolled patients were randomly divided into a training set and a validation set in a 6:4 ratio. The training cohort was used for model development, while the validation cohort served for independent performance evaluation.

To identify prognostic variables associated with overall survival (OS), the least absolute shrinkage and selection operator (LASSO) regression was first applied in the training set to perform dimensionality reduction and variable selection, minimizing overfitting by penalizing redundant covariates. The candidate variables retained after LASSO screening were then incorporated into two machine learning–based survival models: a multivariable Cox proportional hazards regression model and a random survival forest (RSF) model.

The Cox model captured linear associations between variables and OS, whereas the RSF algorithm accounted for nonlinear interactions and complex variable dependencies through ensemble tree learning. Model performance was assessed by concordance index (C-index) and time-dependent receiver operating characteristic (ROC) curves at 1-, 2-, and 3-year intervals.

### Statistical analysis

Continuous variables were compared using the Student’s t-test, and categorical variables were compared using the chi-square test. OS was estimated using the Kaplan-Meier method, and survival differences between groups were assessed with the log-rank test. All analyses and model construction were performed using R software. A two-sided p value <0.05 was considered statistically significant.

## Result

### Baseline characteristics of the study population

A total of 219 patients with locally advanced non-small cell lung cancer (NSCLC) were included in the study. The mean age of the entire cohort was 67.5 ± 9.5 years, and 58.4% of patients were male. The mean tumor size was 54.3 ± 26.0 mm. Most tumors were located in the right lung (65.3%), and the predominant T stage was T4 (42.9%), followed by T3 (26.0%). With respect to nodal involvement, N2 disease accounted for the majority of cases (63.0%). According to the AJCC 8th edition, 45.2% of patients were stage IIIA, 46.6% were stage IIIB, and 8.2% were stage IIIC. Histologically, squamous cell carcinoma was the most common subtype (58.0%), while adenocarcinoma represented 42.0%.

Patients were randomly assigned to the training set (n = 130) and the validation cohort (n = 89). There were no significant differences between the two groups in terms of age, sex, tumor size, location, T stage, N stage, overall stage, or pathological type (all p > 0.05, Table 1), indicating that the baseline characteristics were well balanced between the training and validation cohorts.

**TABLE 1:** Baseline characteristics of the study population.

Variable	Total (N = 219)	Training set (N = 130)	Validation cohort (N = 89)	p-value
Age, year, mean (SD)	67.5 ± 9.50	67.1 ± 9.47	67.9 ± 9.57	0.538
Sex, n (%)				0.893
Female	91 (41.6%)	55 (42.3%)	36 (40.4%)	
Male	128 (58.4%)	75 (57.7%)	53 (59.6%)	
Tumor size, mm, mean (SD)	54.3 (26.0)	54.1 (25.3)	54.5 (27.2)	0.899
Tumor location, n (%)				0.105
Right	143 (65.3%)	91 (70.0%)	52 (58.4%)	
Left	76 (34.7%)	39 (30.0%)	37 (41.6%)	
T stage, n (%)				0.692
T1	29 (13.2%)	17 (13.1%)	12 (13.5%)	
T2	39 (17.8%)	26 (20.0%)	13 (14.6%)	
T3	57 (26.0%)	31 (23.8%)	26 (29.2%)	
T4	94 (42.9%)	56 (43.1%)	38 (42.7%)	
N stage, n (%)				0.661
N0	22 (10.0%)	14 (10.8%)	8 (9.0%)	
N1	25 (11.4%)	13 (10.0%)	12 (13.5%)	
N2	138 (63.0%)	85 (65.4%)	53 (59.6%)	
N3	34 (15.5%)	18 (13.8%)	16 (18.0%)	
Stage, n (%)				0.667
IIIA	99 (45.2%)	62 (47.7%)	37 (41.6%)	
IIIB	102 (46.6%)	58 (44.6%)	44 (49.4%)	
IIIC	18 (8.2%)	10 (7.7%)	8 (9.0%)	
Pathology, n (%)				0.421
Squamous cell carcinoma	127 (58.0%)	72 (55.4%)	55 (61.8%)	
Adenocarcinoma	92 (42.0%)	58 (44.6%)	34 (38.2%)	

### Survival outcomes

**FIGURE 1:**
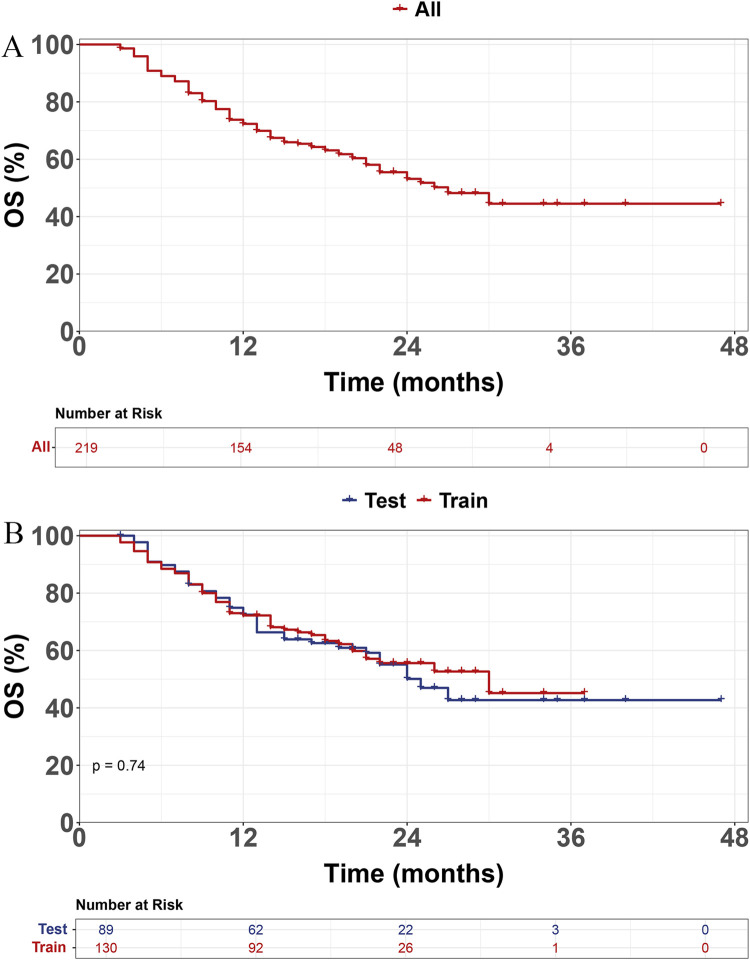
Kaplan-Meier survival curves for all patients **(A)** and for the training and test sets **(B)**.

The training set included 130 patients with 54 deaths, and the validation set included 89 patients with 40 deaths. The median OS (mOS) was 30 (95%CI 21-NA) months in the training set and 25 (95%CI 21-NA) months in the validation set. There was no statistically significant difference in OS between the two cohorts (p = 0.74), as illustrated by the Kaplan–Meier survival curves. The 1-year and 2-year OS were 72% and 53%, respectively (Figure 1).

### AI model construction and validation

**FIGURE 2:**
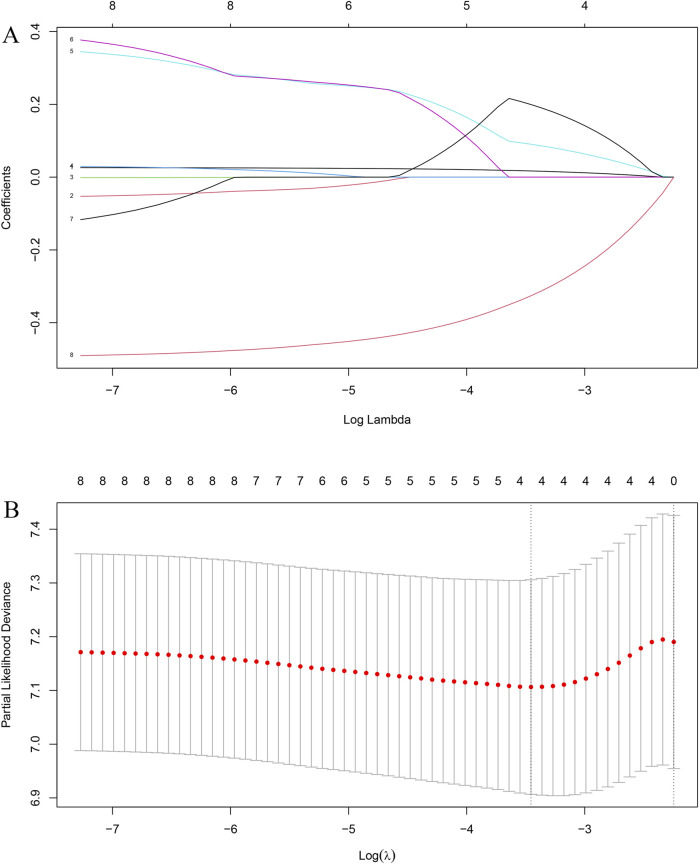
Selection of prognostic variables using LASSO regression. **(A)** LASSO coefficient profiles of clinical variables as a function of the regularization parameter (log λ). **(B)** Ten-fold cross-validation for tuning parameter (λ) selection based on partial likelihood deviance. The optimal λ value (vertical dotted line) was chosen when the model achieved the minimum deviance.

**FIGURE 3:**
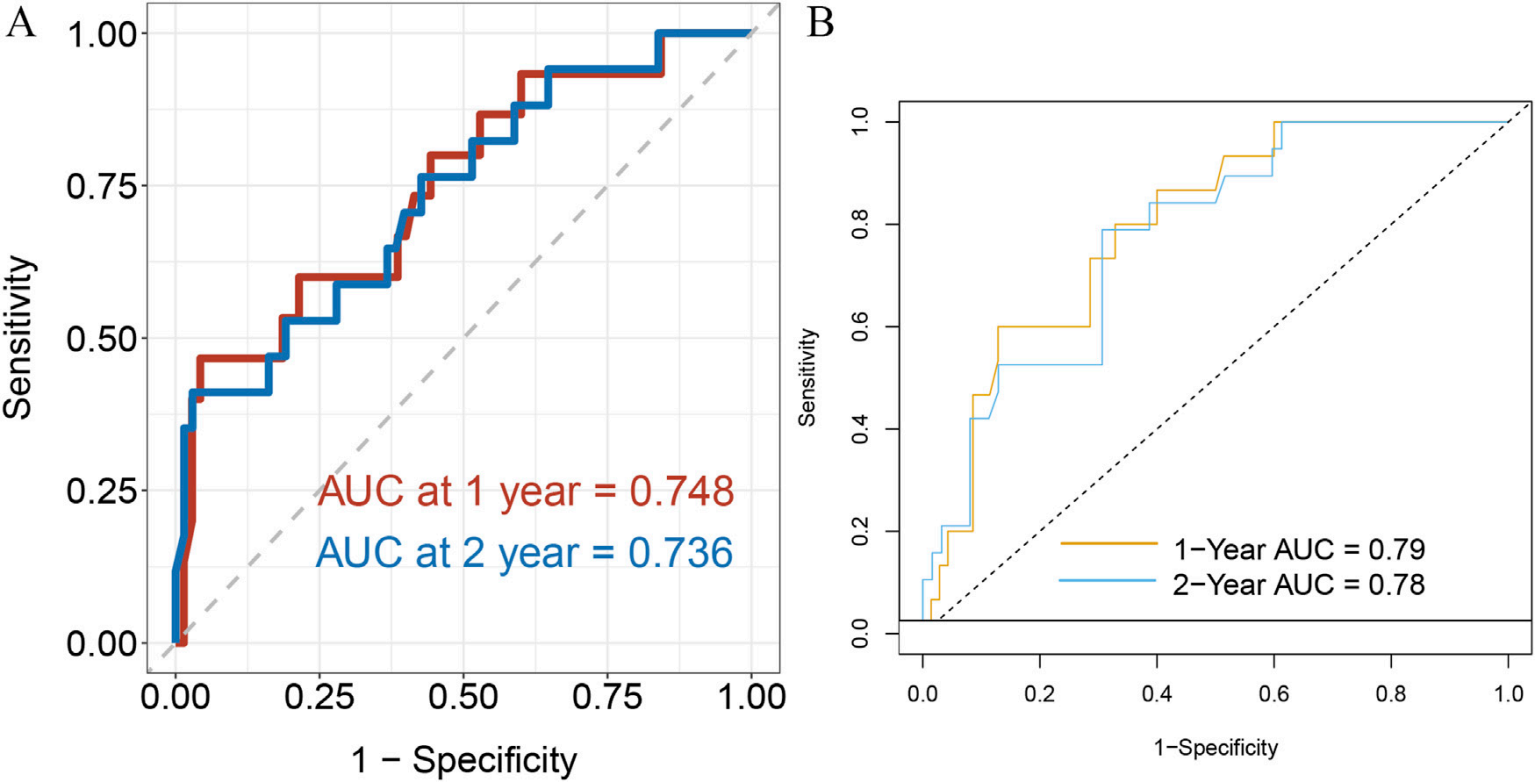
Time-dependent ROC curves for survival prediction models. **(A)** ROC curves of the multivariable Cox model for predicting 1-year and 2-year overall survival (OS), with AUCs of 0.748 and 0.736, respectively. **(B)** ROC curves of the random survival forest (RSF) model for predicting 1-year and 2-year OS, with AUCs of 0.79 and 0.78, respectively.

LASSO regression was first performed to identify prognostic variables associated with OS, and four variables—Age, T stage, Stage, and Pathology—were selected for further modeling (Figure 2). In the training set, multivariate Cox proportional hazards analysis confirmed that Stage and Pathology were independent prognostic factors for OS. The concordance index (C-index) of the Cox model in the training cohort was 0.62. The results of the multivariate Cox regression analysis in the training set are presented in Table 2. Significant independent prognostic factors for OS included Stage and Pathology. Specifically, patients with Stage IIIC had a higher hazard ratio (HR = 2.83, 95% CI = 1.12–7.18, p = 0.028) compared to those with Stage IIIA, while Pathology (AD vs. SCC) also showed a significant association with OS (HR = 0.55, 95% CI = 0.30–0.98, p = 0.043). In the validation cohort, time-dependent ROC analysis demonstrated good predictive performance, with AUCs of 0.748 and 0.736 for 1-year and 2-year OS, respectively (Figure 3A).

**TABLE 2:** Multivariable Cox proportional hazards analysis in training set.

Variable	Category/Mean ± SD	HR (95% CI)	P
Age	Mean ± SD	1.00 (0.97–1.04)	0.777
T Stage	T1	Reference	
	T2	1.27 (0.45–3.59)	0.656
	T3	1.28 (0.45–3.63)	0.647
	T4	1.29 (0.50–3.31)	0.599
Stage	IIIA	Reference	
	IIIB	0.93 (0.47–1.84)	0.833
	IIIC	2.83 (1.12–7.18)	0.028
Pathology	SCC	Reference	
	AD	0.55 (0.30–0.98)	0.043

An additional RSF model was then constructed in the training set to further enhance predictive performance. This model achieved a higher C-index of 0.67 in the training cohort. In the validation cohort, the time-dependent ROC curves demonstrated strong discrimination, with AUCs of 0.79 and 0.78 for 1-year and 2-year OS, respectively (Figure 3B).

**FIGURE 4:**
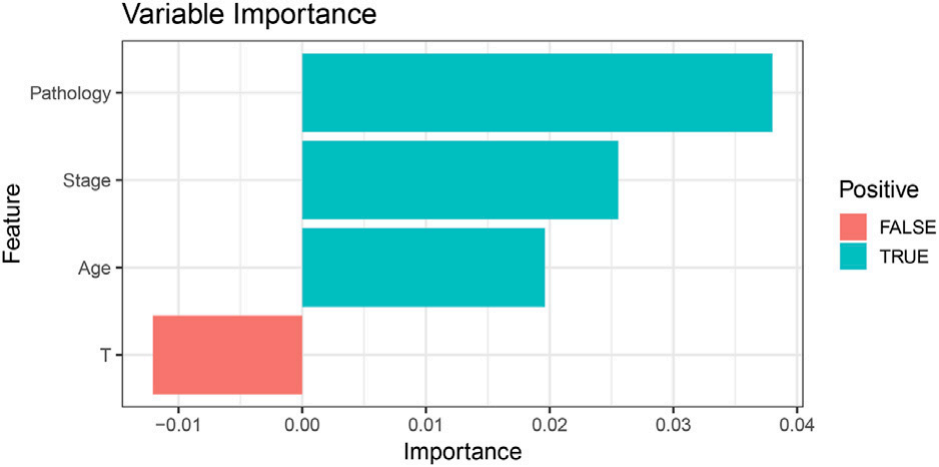
Variable importance ranking in the RSF model. The random survival forest (RSF) model identified Pathology and Stage as the most influential predictors of overall survival, followed by Age and T stage. Positive values indicate variables contributing to improved prediction accuracy.

**FIGURE 5:**
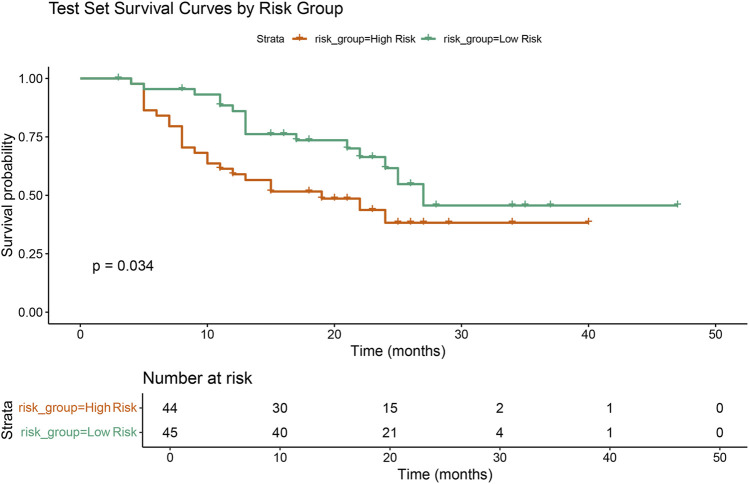
Kaplan–Meier survival curves by RSF-based risk groups. Kaplan–Meier curves of the validation set showing overall survival (OS) between the high-risk (orange) and low-risk (green) groups defined by the RSF model. Patients in the high-risk group exhibited significantly poorer survival compared with those in the low-risk group (p = 0.034).

In the RSF model, the variable importance plot revealed that Pathology and Stage were the two most influential factors contributing to survival prediction (Figure 4). Based on the RSF-derived prognostic scores, a risk score was calculated for each patient. Using the median risk score as the cutoff value, patients were stratified into high- and low-risk groups. Kaplan–Meier survival analysis showed that patients in the high-risk group had significantly poorer OS compared with those in the low-risk group (p < 0.034, Figure 5), indicating that the RSF-based AI model effectively distinguished prognosis among individuals with locally advanced NSCLC.

## Discussion

This study established machine learning–based survival prediction models to explore prognostic determinants in patients with locally advanced NSCLC who underwent concurrent chemoradiotherapy followed by consolidation immunotherapy. Through integration of clinical variables and machine-learning algorithms, we sought to construct a data-driven framework to improve individualized outcome prediction and support evidence-based treatment planning in the precision medicine era. By comparing conventional Cox regression with the RSF algorithm, we demonstrated that AI-based models can more accurately characterize patient heterogeneity and provide refined prognostic stratification.

Over the past decade, therapeutic strategies for locally advanced NSCLC have undergone substantial transformation. CCRT remains the standard of care, offering improved local control compared with sequential regimens (Herbst et al., 2022). The introduction of ICIs, particularly consolidation therapy with PD-1/PD-L1 inhibitors after CCRT, has revolutionized outcomes, with phase III trials such as PACIFIC confirming prolonged survival and durable tumor control (Spigel et al., 2022; Faivre-Finn et al., 2021). However, significant variability in clinical benefit persists, driven by differences in tumor biology, immune activation, and treatment tolerance. In this context, predictive tools that can stratify patients by expected response are urgently needed. Our study’s strength lies in leveraging AI to integrate multiple clinical parameters, enabling a quantitative, objective, and reproducible method to forecast prognosis—an advantage over traditional empirical or univariate clinical assessment.

The emergence of AI has redefined predictive modeling in oncology (Prelaj et al., 2024). Traditional Cox regression models, although widely used, rely on the proportional hazards assumption and are limited to linear relationships, potentially overlooking complex interactions among variables. In contrast, AI-based methods such as RSF can learn nonlinear dependencies, handle multicollinearity, and incorporate high-dimensional data without prespecified assumptions (Honvoh et al., 2022; He et al., 2020). In our study, the RSF model achieved higher discriminative performance than the Cox model (C-index 0.67 vs. 0.62), along with superior time-dependent AUCs at both 1 and 2 years. These results underscore the ability of AI to reveal hidden structures within clinical datasets, offering a more flexible and adaptive approach to survival prediction. Moreover, RSF’s ensemble learning mechanism minimizes overfitting and enhances model stability, making it particularly suitable for heterogeneous cancer populations (Tian et al., 2023; Zeng et al., 2022; Hernández-Boluda et al., 2025).

Four variables—Age, T stage, Stage, and Pathology—were identified by LASSO regression as potential prognostic indicators. Among them, Stage and Pathology remained independent predictors in multivariate Cox analysis. Advanced tumor stage reflects increased tumor burden, greater nodal involvement, and diminished local control after chemoradiation, all of which translate to shorter survival (Evison and AstraZeneca UK Limited, 2020). Histological subtype also played a critical role: squamous cell carcinoma and adenocarcinoma differ not only in genomic alterations but also in immune microenvironmental profiles, influencing radiosensitivity and response to immunotherapy (Giaccone and He, 2023). Although Age and T stage did not retain independent statistical significance, their biological and clinical relevance should not be overlooked. Age may indirectly affect prognosis through performance status and treatment tolerance, while T stage contributes to tumor hypoxia and local progression (Zhang et al., 2024; Cai et al., 2022). Collectively, these findings confirm that survival in NSCLC results from the interplay of tumor burden, pathological features, and host-related factors.

From a clinical perspective, the AI-derived RSF model enables individualized risk scoring and dynamic patient stratification. By using the median risk score as a cutoff, the model effectively distinguishes between high-risk and low-risk groups, with high-risk patients demonstrating significantly worse overall survival. This ability to provide quantitative risk assessments has important practical implications: clinicians can identify patients who may benefit from intensified surveillance, adjuvant therapies, or inclusion in clinical trials, while low-risk patients can avoid unnecessary treatments. Additionally, integrating such predictive systems into multidisciplinary tumor boards could support decision-making related to radiotherapy dose adjustments, immunotherapy sequencing, and collaborative treatment planning. As electronic health records and radiotherapy planning data continue to digitalize, the clinical adoption of AI tools is both feasible and timely (Wang et al., 2024; Xu et al., 2025; Song et al., 2025).

Despite its promising results, this study has several limitations. First, it was retrospective in design and included a relatively small sample from two institutions, which may introduce selection and information bias. Second, only clinical variables were analyzed; imaging radiomics, genomic, or immunological biomarkers were not incorporated, limiting biological interpretability and model generalizability. Third, while internal validation confirmed predictive robustness, external validation using independent prospective cohorts is required before clinical implementation. Finally, the model operates in a static framework and does not yet support real-time learning or continuous updating based on longitudinal outcomes—an aspect that future adaptive AI systems could address.

In conclusion, this study developed and validated AI-based prognostic models for locally advanced NSCLC patients treated with chemoradiotherapy followed by consolidation immunotherapy. The RSF model demonstrated improved performance over the traditional Cox regression model, offering better accuracy and interpretability in survival prediction and risk stratification. These findings suggest the potential of AI in oncology, supporting the integration of clinical data analytics with personalized care. Future research incorporating AI with radiomic and molecular biomarkers, along with large-scale multicenter prospective validation, will be essential to explore the full potential of precision immuno-radiotherapy and to further develop intelligent prognostic modeling for clinical practice.

## Data Availability

The original contributions presented in the study are included in the article/supplementary material, further inquiries can be directed to the corresponding author.
